# Supplementary material for: Treatments and interventions addressing chronic somatic pain in torture survivors: A systematic review
Source: PLOS Glob Public Health. 2024 Mar 28;4(3):e0003070. doi: 10.1371/journal.pgph.0003070 (PMC10977680; doi:10.1371/journal.pgph.0003070)
Supplement: S1 Table — (DOCX) [file pgph.0003070.s002.docx]

**S1 Table**

# **Evidence Project risk of bias tool completed for the review**

| **Study** | **Cohort** | **Control or comparison group** | **Pre/post intervention data** | **Random assignment of participants to the intervention** | **Random selection of participants for assessment** | **Follow-up rate of 80% or more** | **Comparison groups equivalent on sociodemographics** | **Comparison groups equivalent at baseline on outcome measures** |
| --- | --- | --- | --- | --- | --- | --- | --- | --- |
| Dibaj 2017 | Yes | No | Yes | NA* | No | No | NA | NA |
| Dix-Peek 2018 | Yes | Yes | Yes | No | No | No | No | No |
| Jorgensen 2015 | Yes | No | Yes | NA | No | Yes | NA | NA |
| Kim 2015 | Yes | Yes | Yes | Yes | No | Yes | Yes | NR |
| Nordin 2019 | Yes | No | Yes | NA | No | No | NA | NA |
| Phaneth 2014 | Yes | No | Yes | NA | No | Yes | NA | NA |
| Northwood 2020 | Yes | Yes | Yes | Yes | No | Yes | Yes | Yes |
| Gamble 2020 | Yes | Yes | Yes | Yes | No | Yes | Yes | Yes |
| Neuner 2010 | Yes | Yes | Yes | Yes | No | Yes | Yes | Yes |
| Wang 2016 | Yes | Yes | Yes | Yes | No | Yes | Yes | Yes |

**NA* not applicable
