# Supplementary material for: Treatments and interventions addressing chronic somatic pain in torture survivors: A systematic review
Source: PLOS Glob Public Health. 2024 Mar 28;4(3):e0003070. doi: 10.1371/journal.pgph.0003070 (PMC10977680; doi:10.1371/journal.pgph.0003070)
Supplement: S2 Table — (DOCX) [file pgph.0003070.s003.docx]

**S2 Table**

# **Characteristics of excluded studies (ordered by study ID)**

Studies excluded (n = 47)

Not in English (n = 3)

Incorrect outcomes (n = 15)

Irrelevant intervention (n = 11)

Incorrect study design (n = 16)

Irrelevant patient population (n = 2)

| **Study** | **Reason for exclusion** |
| --- | --- |
| Adenauer 2011 | Irrelevant intervention |
| Alayarian 2009 | Incorrect study design |
| Allden 1998 | Incorrect study design |
| Apitzsch 1996 | Not in English |
| Bager 2018 | Incorrect outcomes |
| Baird 2017 | Incorrect study design |
| Baird 2017 | Duplicate |
| Başoğlu 2004 | Irrelevant intervention |
| Bass 2016 | Incorrect outcomes |
| Bass 2012 | Incorrect outcomes |
| Bass 2013 | Irrelevant intervention |
| Berthold 2020 | Incorrect study design |
| Bichescu 2007 | Irrelevant intervention |
| Bolton 2014 | Incorrect outcomes |
| Brogan 2017 | Incorrect outcomes |
| Buhmann 2015 | Incorrect outcomes |
| Carlsson 2005 | Incorrect outcomes |
| Carlsson 2010 | Incorrect outcomes |
| Chang 2018 | Incorrect outcomes |
| Dee 2022 | Irrelevant patient population |
| Drozdek 2014 | Irrelevant intervention |
| Esala 2017 | Incorrect outcomes |
| Fernandez 2001 | Irrelevant intervention |
| Fischman 1990 | Incorrect study design |
| Grodin 2008 | Incorrect study design |
| Halvorsen 2010 | Incorrect outcomes |
| Hamid 2019 | Incorrect study design |
| Hensel-Dittmann 2011 | Irrelevant intervention |
| Keshk 2021 | Incorrect study design |
| Knaevelsrud 2007 | Incorrect study design |
| Larsen 1987 | Incorrect study design |
| Lie 1996 | Not in English |
| Looi 1996 | Irrelevant intervention |
| Morina 2010 | Not in English |
| Morris 1993 | Irrelevant patient population |
| Munczek 1998 | Incorrect study design |
| NCTTP, 2015 | Incorrect study design |
| Nieves-Grafals 2001 | Incorrect outcomes |
| Nilsson 2021 | Incorrect outcomes |
| Norredam 2005 | Incorrect study design |
| Pabilonia 2010 | Incorrect outcomes |
| Precin 2011 | Irrelevant intervention |
| Puvimanasinghe 2016 | Irrelevant intervention |
| Reid 1990 | Incorrect study design |
| Sahdev 2020 | Irrelevant intervention |
| Stammel 2017 | Incorrect outcomes |
| Weine 1995 | Incorrect study design |
