# Supplementary material for: Treatments and interventions addressing chronic somatic pain in torture survivors: A systematic review
Source: PLOS Glob Public Health. 2024 Mar 28;4(3):e0003070. doi: 10.1371/journal.pgph.0003070 (PMC10977680; doi:10.1371/journal.pgph.0003070)
Supplement: S1 Text — (DOCX) [file pgph.0003070.s004.docx]

**S1 Text**

# **Search strategy**

Pubmed (02/21/23)

("Torture"[Mesh] OR tortur*[tiab]) AND ("Nociceptive Pain"[Mesh] OR "somatic pain" OR pain[tiab] OR "Quality of Life"[Mesh] OR "quality of life" OR "distress*" OR "disorder*") AND (treat*[tiab] OR reduc*[tiab] OR interven*[tiab])

Medline (02/21/23)

1.            exp Torture/

2.            tortur*.ti,ab.

3.            1 or 2

4.            exp Nociceptive Pain/

5.            ((nociceptive or somatic or tissue) adj5 pain*).ti,ab.

6.            exp "Quality of Life"/

7.            ((qualit* adj2 life) or distress* or disorder*).ti,ab.

8.            4 or 5 or 6 or 7

9.            (treat* or reduc* or interven* or improv*).ti,ab.

10.          3 and 8 and 9

EMBASE (02/21/23)

1.            exp torture/

2.            tortur*.ti,ab.

3.            1 or 2

4.            exp         nociceptive pain/

5.            ((nociceptive or somatic or tissue) adj5 pain*).ti,ab.

6.            exp         "quality of life"/

7.            ((qualit* adj2 life) or distress* or disorder*).ti,ab.

8.            4 or 5 or 6 or 7

9.            (treat* or reduc* or interven* or improv*).ti,ab.

10.          3 and 8 and 9
