# Supplementary material for: Treatments and interventions addressing chronic somatic pain in torture survivors: A systematic review
Source: PLOS Glob Public Health. 2024 Mar 28;4(3):e0003070. doi: 10.1371/journal.pgph.0003070 (PMC10977680; doi:10.1371/journal.pgph.0003070)
Supplement: Attachment — Submitted filename: Response to Reviewers.pdf [file pgph.0003070.s005.pdf]

Weill Cornell Medicine
Anesthesiology

Gunisha Kaur, MD, MA
Medical Director, Weill Cornell Center for Human Rights
Associate Professor of Anesthesiology
Founding Director, Human Rights Impact Lab
Co-Medical Director, Weill Cornell Center for Human Rights
Weill Cornell Medicine/New York-Presbyterian Hospital

February 8, 2024

Dear Editor,

Thank you for your review of our manuscript (PGPH-D-23-02521) entitled, "Treatments and interventions addressing chronic somatic pain in torture survivors: a systematic review." Please see our attached revised manuscript, marked up manuscript, figures, and supplementary files, as well as our responses to editors and reviewers below. We thank you for your review of our resubmission and hope that our adjustments are helpful. If there are further edits or information the authors can provide, we would be glad to do so.

EDITOR COMMENTS

Please review your reference list to ensure that it is complete and correct. If you have cited papers that have been retracted, please include the rationale for doing so in the manuscript text, or remove these references and replace them with relevant current references. Any changes to the reference list should be mentioned in the rebuttal letter that accompanies your revised manuscript. If you need to cite a retracted article, indicate the article's retracted status in the References list and also include a citation and full reference for the retraction note.

Thank you for this comment. *No retracted studies were included in our paper*. Rather, the inclusion of a retracted study is one of our critiques of the only other, most recent systematic review of pain interventions in torture survivors. The reference list has been checked for completeness and correctness. The following citations were added: 7, 8, 9, 18, 19, 32, 44, 45, 46.

REVIEWER ONE COMMENTS

The authors report the results of arduous research, either for the scarce literature available on the issue "torture", or for the need to emphasize the consequences of such trauma, not only psychologically but also in terms of somatic pain, a topic likely under evaluated or rarely addressed. Papers not written in English were not taken into account, and this could be a fault of the paper. Nevertheless, the paper is well written and the results are properly analyzed. References are appropriate and numerous.

We thank the reviewer for their kind comments. We believe this work will fill a major gap in the literature around the known effectiveness of chronic pain treatments for torture survivors.

Limited effectiveness and variable outcomes were observed in evaluating therapies for chronic pain following tortures. Accordingly, this paper is a clear stimulus to further research in order to highlight the problem and optimise existing therapeutic interventions.

Thank you for this comment. We agree and believe strongly that this paper represents the responsibility to conduct further investigations into treatments for chronic somatic pain after torture.

REVIEWER TWO COMMENTS

I would like to extend my thanks for getting the chance to review this spectacular paper. I truly believe the paper has strong qualities, but I will focus on the areas for improvement. I have put forth my suggestions below for the

consideration of the authors. I hope you will address the suggestions before endorsement of publications, especially those in the discussion section.

Introduction. The introduction section has a truly informative flow and you have justified the need for the study without a doubt. You have mentioned how the pain experience of the torture survivors is complex, if you could explain on that a little bit further in an additional paragraph, where you can also extend by outlining possible interventions and treatments currently on practice, irrespective of the evidence meagerness, you will definitely set up your readers in the best position. Line 60 - the previous comment is best entertained after this paragraph. Thank you for this thoughtful comment. While being mindful of space limitations, we have added content where we outline the complexities of chronic somatic pain after torture, and the possible interventions and treatments that are currently in practice. We agree that this enhances the reader experience. This adjustment is reflected on Pages 3-4, Lines 54-82.

Methods. Search strategy and selection criteria. I truly believe you are addressing an area with critical evidence need. So is it possible to consider meta-analytic approaches to quantify the effect size of the interventions? I believe, if that is possible, your review would be a milestone in pain management of torture survivors. If possible, please go for it, even though the studies may prove to be highly heterogeneous.
We thank the reviewer for this comment and absolutely agree that a meta-analysis would have been the ideal outcome. In fact, this is what we initially set out to do, and indeed we did attempt a meta-analysis. However, because of both the exceedingly small number of studies in this space *and* the quality of the studies *and* the heterogeneity of the studies, we were unable to analyze with any rigor. Our group believes strongly that more *high quality*, rigorous data are needed in the field of refugee health research, and we have built our laboratory, which is one of the only federally funded labs for refugee health research in the U.S., around this concept of rigor, reproducibility, and excellence. As much as we would like to be able to contribute a meta-analysis in this space, we do not want to contribute low-quality data, given that we are limited by the study-specific issues described above. Rather, we would use this as an opportunity to highlight the deficiency in the literature and advocate for further rigorous research in the area. We have adjusted the manuscript in this way, which is reflected on Page 19, Lines 376-383. Thank you again for this comment!

Line 91 - it's better to put your search strategy domains in standard PICO format to ensure clarity and comparison with future studies.
Thank you for this helpful suggestion. We have adjusted the search strategy domains to be in the standard PICO format. This adjustment is reflected on Page 5, Lines 122-123.

Study selection.
In addition to assessing eligible studies, based on inclusion and criteria, through full text review, please also include if you have taken any quality assessments.
Thank you for this feedback. On the guidance of our expert librarian, we utilized the Evidence Project Risk of Bias Tool for quality assessment of the studies. This tool assesses randomized controlled trials (RCTs), non-RCTs, observational, and quasi-experimental studies. The completed tool for this review is available in Appendix 3. We have clarified this in the text on Page 6, Lines 151-154 and Page 17, Lines 327-328.

Line 106 - since your study focused on pain after torture as clearly shown in you inclusion criteria, it will be prudent to discuss a little bit on peculiarities of chronic pain in those with torture as compared to other common causes of chronic pain. This will further justify the need for considering pain uniquely in torture survivors.
Thank you for this helpful suggestion. We agree and have made this adjustment in the description of chronic somatic torture pain in the Introduction. Please see Pages 3-4, Lines 54-78.

Department of Anesthesiology 525 East 68th Street, Box 124 New York, NY 10065
T. 212.746.2461 | E. gus2004@med.cornell.edu

Data analysis.
The previous comment on meta-analytic approaches is a kind plea for possible reconsideration. But please note, I fully accept your justifications.
Thank you again for this suggestion. We could not agree more with you that a meta-analysis would be ideal. Unfortunately, for the reasons listed above, we do not believe that this would be our best contribution to the literature.

Results.
Definitions of torture and pain in the studies' populations. Please discuss a little on this torture-like experiences on the introduction and their possible implication on the chronic pain experiences, this would best position your readers.
Thank you for this suggestion. We have made this addition in the Introduction, alongside the reviewer's helpful comments about the other additions related to the nature of chronic torture pain, as well. Please see Pages 3-4, Lines 54-78.

Table 2. Intervention Description and Overall Effect on Pain Outcomes. It's better to keep some kind of order to the list of studies here and in the previous descriptive table of studies, either sample size, alphabetically, or just keep the orders of the two tables similar.
Thank you for this wise suggestion. In line with the published literature, we have revised both Tables to be in alphabetical order.

I would also strongly suggest the inclusion of additional column to clearly put the different intervention and control groups, or just the intervention group for those with no control group. Because I seem to get lost between which studies were randomized or not so better include it here in a clear manner just for simplicity.
Thank you for this helpful suggestion. We agree and have added this column.

Northwood 2020 - IPCM? - Please write this acronym fully, since you used it for first time here. Dix-Peek 2018 - CSVR framework - also consider writing out this acronym.
Thank you. Both of these have been written out fully. Please see Table 2.

Discussion. I truly believe you could work on the depth of your discussion, considering the "dearth of knowledge" existing in this research question. I suggest few ideas for you to discuss on in this section.
Thank you for this very helpful suggestion and outline of items to add. While being mindful of space restrictions, we have added all of these elements and believe that the Discussion is far more robust with the reviewer's helpful guidance. Please see below for location of changes.
- Discuss on the intervention approach which had consistently shown improvement of pain outcome, speculate about why the approach would have been effective compared to the others triangulating with the current pathophysiological or psychological understanding. Please see Pages 17-18, Lines 331-349.
- Also discuss on the possible impact of pain measurement tools, those used by most studies, sensitivity in detecting impact of interventions. Discuss this with context of clinical practice and its implication for those treating these patients. Please see Page 18, Lines 350-358.
- Also sift targeted recommendations for practices on patient management, though inconclusively. Please see Page 18, Lines 347-358.
- Also elaborate further on possible pitfalls to avoid on future study designs you have recommended on the last paragraph. Please see Page 19, Lines 376-394.

Department of Anesthesiology 525 East 68th Street, Box 124 New York, NY 10065
T. 212.746.2461 | E. gus2004@med.cornell.edu

REVIEWER THREE COMMENTS.
Overall, this is a methodologically rigorous systematic review of an important topic, i.e., interventions addressing chronic somatic pain in torture survivors. It is well researched and well written and should be accepted with minor revisions; focused mostly on presentation of the information contained within the second table. Specifically, I would suggest the following:
We thank the reviewer for this kind comment. We agree that this is an incredibly needed, increasingly important area of scientific research. We are glad to contribute to closing the gap in the literature.

Introduction is thin; would consider adding some more information about the most common modalities subsequently mentioned and their demonstrated efficacy in other special populations. Would also mention some of the challenges and comparative strategies for assessing/measuring pain including across cultures.
Thank you for this comment. We agree and have made adjustments to increase these descriptions, while being mindful of space restrictions as per journal guidelines. Related, relevant content has also been added to the Discussion. Please see Pages 3-4, Lines 64-84, and Pages 17-19, Lines 322-394.

Posttraumatic Stress Disorder should be spelled out at first mention with (PTSD) alongside, then referred to by abbreviated name moving forward.
Thank you. We have made this adjustment.

Page 9 lines 203-204: re: "All trials excluded participants if they presented with a psychiatric condition" - this is a bit confusing given the prevalence of PTSD in this population; is this referring to an acute psychiatric emergency?
Thank you for this comment. Yes, you are correct that this was in reference to acute or confounding psychiatric condition, such as psychosis. We have made the adjustment in the paper. Please see Page 10, Lines 241-242.

Page 13 lines 227-228: re: "two studies utilized Narrative Exposure Therapy (NET) in some capacity within the intervention" - this is confusing as NET is mentioned in four different interventions in Table 1 (Neuner, Northwood, Dibaj, and Dix-Peek).
Thank you for this question. Two of the *Pre-post-test studies* utilized NET. The other studies astutely listed by the reviewer were RCTs, and are described in the section above the Pre-post-test section. We have adjusted the language for clarity. Please see Page 13, Line 268.

Table 2: I recommend splitting into two tables as follows:
-- One table should include definitions of all individual components/modalities mentioned (i.e., NET, CBT, Sensorimotor Psychotherapy, BF, PET, PT, motivational interviewing, case management, DBT, TT, MFR, etc.) independent of the studies. You could add a column mentioning which studies it was included in (e.g., for NET, Neuner, Northwood, Dibaj, and Dix-Peek) +/- the percentage of studies which employed it to get a sense of how commonly it was being used.
-- A separate second table should then describe exactly what each of the interventions did, engaging abbreviations from preceding table. For example, for Dibaj 2017, NET and PT would be defined in initial table whereas the second table would describe intervention as "20 sessions of NET (90 min each) and 10 sessions of PT (60 min each). For the second table, results should include which measures were being used (e.g., for Dibaj, NOSF-MISS).
Thank you for this helpful comment. With the reviewer's suggestion, we attempted to make two Tables – one with a description of the treatments, and one with the studies and which they utilized. However, this did not work and became incredibly complicated. The reason for this is that each study utilized a variation or flavor of a stated intervention (e.g., there is no standardized definition of "case management" or "physiotherapy" or many

Department of Anesthesiology 525 East 68th Street, Box 124 New York, NY 10065
T. 212.746.2461 | E. gus2004@med.cornell.edu

other treatments; they are patient or study-specific, which then required us to describe them in the second table, and required the reader to then match it to the intervention abbreviation in the first table). For example, Dix-Peek utilized trauma-focused Cognitive Behavioral Therapy, Gamble used a unique combination of physiotherapy, Kim deployed particular exercises in their Complex Manual Therapy. Because there is such variation in the study-specific uses of therapies, we found that listing the details alongside the study decreases the strain on the reader (i.e., otherwise they would have to match up cluttered information from two separate tables, because there is no standard definition of specific interventions that is used universally for all studies). In the spirit of the reviewer's suggestion, we did streamline the abbreviations in the Table caption, but even this was quite limited because there is so much internal variation in how therapies are deployed among studies. We hope this is satisfactory, but if the reviewer believes that additional improvements can be made and can suggest how, we would gladly reapproach this. Please see our adjustments on Table 2.

Page 17 lines 283-284: re: "the quality of included studies was generally good) - how did you make this assessment? Did you consider using a Mixed Methods Appraisal Tool (MMAT) to formally assess quality? https://www.mcgill.ca/familymed/research/projects/mmat
Thank you for this comment. As recommended by our expert librarian, we utilized the Evidence Project Risk of Bias tool to assess the quality of the studies. The completed tool for this review is available in Appendix 3. This has been clarified in the paper. Please see Page 6, Lines 151-154 and Page 17, Lines 327-330.

Page 17 lines 292-294: re: "Given the exponential rise of forcibly displaced refugee populations – currently at one in ten people" - what is 1 in 10 referring to? Surely not overall number of displaced individuals worldwide (108 million, or approximately 1.35 per 100 total world population).
Thank you for this! We have adjusted this error – we had intended for it to say one in 100, in line with the most recent report from the United Nations High Commissioner for Refugees, which we have cited. Please see Pages 18-19, Lines 363-374.

Discussion is quite limited; would expand significantly and make clear what the take-home points should be for readers (consider adding Conclusions section).
Thank you for this helpful feedback, which is similar to Reviewer 2's input. We have made significant adjustments to the Discussion to make it more robust. Please see Pages 17-19, Lines 322-394.

Thank you for your consideration of our revised manuscript with comments. If there are further adjustments the authors can make to the paper, we would be glad to consider any feedback the editor or reviewers may have.

Sincerely,

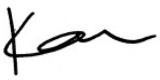

Gunisha Kaur, MD, MA

Department of Anesthesiology 525 East 68th Street, Box 124 New York, NY 10065

T. 212.746.2461 | E. gus2004@med.cornell.edu
